# Magnitude and associated factors of delirium among patients attending emergency department at Jimma medical center, Jimma, southwest Ethiopia, 2022

**DOI:** 10.1186/s12888-022-04408-x

**Published:** 2022-12-02

**Authors:** Bethelhem Sileshy, Hailemariam Hailesilasiie, Yonas Tesfaye, Henok Ababu

**Affiliations:** 1 Department of Nursing, College of Health and Medical Science, Wolkite University, Wolkite, Ethiopia; 2 Department of Psychiatry, Faculty of Medicine, Jimma University, Jimma, Ethiopia; 3 Department of Psychiatry, College of Health and Medical Science, Dilla University, Dilla, Ethiopia

**Keywords:** Delirium, Emergency patients, Associated factors, Jimma, Ethiopia

## Abstract

**Background:**

Delirium is a severe neuropsychiatric condition that occurs frequently in all medical settings. It has been associated to serious consequences like higher mortality, institutionalization, and longer hospital stays. Delirium is missed in emergency rooms in 57% to 83% of patients, despite its frequent incidence and detrimental repercussions.

**Objectives:**

The purpose of this study was to determine the prevalence and contributing causes of delirium in patients who visited the emergency room at Jimma Medical Center in Jimma, southwest Ethiopia, in 2022.

**Methods:**

From August 1 through September 30, 2022, a cross-sectional study was undertaken at a hospital. The study enrolled 422 participants, who were chosen through a systematic random sampling. The Richmond Agitation Sedation Scale (RASS) was used to evaluate different subtypes of delirium and level of arousal. The Confusion Assessment Method (CAM) was used to determine the presence or absence of delirium. Epi Data V3.1 was used to enter the data, and Version 20 of the Statistical Package for Social Scientists was used to export it (SPSS V20). Bivariate and multivariable logistic regressions were performed to identify the related factors. Variables with a *p*-value of less than 0.05 were considered to be significant.

**Result:**

26.6% of participants (*n* = 107) were found to have delirium. Alcohol use (AOR = 3.6, 95% CI (2.5–8.1), visual impairment (AOR = 2.34, 95% CI (1.89–3.68), frequent admission (AOR = 3.47, 95% CI (1.24–7.34), bladder catheterization (AOR = 1.4, 95% CI (1.21–2.89), and benzodiazepine exposure (AOR = 1.5, 95% CI (1.01–2.3) had a significant association with delirium.

**Conclusion:**

According to this study, delirium was very common among patients in the emergency room. Benzodiazepine exposure, numerous admissions, visual impairment, current alcohol consumption, bladder catheterization, and frequent admissions all significantly increased the risk of delirium. To address identifiable causes and enhance patients’ health outcomes, early recognition is crucial.

## Introduction

Delirium is among the most prevalent neurocognitive disorders, with a sudden onset, rapidly changing cognitive decline, and impairment of conscious experience which is characterized by abnormalities of orientation, memory, communication skills, reasoning, awareness, motor behavior, sleep–wake pattern, and abnormal attention as the key cognitive disturbance that is not better explained by pre-existing, identified, or other progressing neurobiological disorders [1, 2].

Delirium develops suddenly and swings over a day [3]. Even though its manifestation is often linked to hyperactive delirium symptoms (instability, restlessness), there are two distinct subgroups, notably mixed and hypoactive delirium [4]. Lack of energy, diminished alertness, and indifference are the hallmarks of hypoactive delirium, while mixed delirium combines elements of both delirium subtypes [3]. Despite having negative clinical effects, delirium is often treatable [5, 6].

It is linked to a number of negative consequences, such as longer hospital stays, higher death rates, and institutionalization, all of which are particularly concerning in low-income nations [7, 8]. Despite its widespread prevalence and detrimental effects, emergency physicians miss delirium in 57% to 83% of cases [9, 10]. Compared to patients whose delirium is picked up by emergency physicians, there is some evidence to suggest that missing delirium in the emergency unit portends increased risk [9]. In previous researches, delirium was prevalent in the range of 9–35% among medical inpatients in the United States [11], the United Kingdom [12], Australia [13], Spain [14], and Sub-Saharan Africa [15, 16].

The goal of the study was to identify the prevalence of delirium and its determinants among patients using Jimma Medical Center’s emergency room. Therefore, studying delirium in emergency patients might be valuable in identifying those who need early intervention the most, thus lowering the negative effects of this condition.

## Methods

### Study setting

The study was conducted in Jimma medical center, emergency department, which is found in Jimma town, Oromia regional state in southwest Ethiopia which is 352 km away from the capital. It provides services for about 15 million populations in the south-west catchment area. Around 2000 patients attend the emergency department per month.

### Eligibility criteria

All patients attending the emergency department at Jimma medical center during the study period were included in the study. Patients who are comatose with RASS score of -4 or -5 and patients with severe dementia were excluded from the study.

### Sample size and sampling technique

Single population proportion formula was used to determine sample size and a *p*-value of 50% was taken since there is no study conducted on the area of interest specific to the study setting and 95% confidence interval, a margin of error of 5%, a non-response rate of 10% was used to get the total sample size of 422. A systematic Random sampling technique was employed to select study subjects. Selection skip interval (k) was calculated by taking the total of patients attending the emergency department per month 2000(N) to the sample size (n) 422 = N/n, k = 2000/422 =  = 4.74 = 5, so the participants were selected every 5th interval, who visited the emergency department during the data collection period. The first respondent was selected by lottery method and the next respondent was chosen at regular intervals.

### Study variables

#### Dependent variable

- Delirium (yes/no)

#### Independent variables

❖ Demographic factors:- Age, sex, religion, marital status, ethnicity, educational status, and economic status
❖ Substance use:- Alcohol use, Khat use, Cigarette smoking
❖ Chronic physical illness:- Heart diseases, Diabetes Mellitus, and other chronic physical illnesses
❖ Physical Impairment:- Visual, Hearing, and Cognitive
❖ Medication-related:- Previous or current anticholinergic, benzodiazepines, polytherapy, and antipsychotics use
❖ Hospital-related factors:- Bladder catheterization, Intravenous fluid, the severity of illness, and frequent Admissions

#### Operational definition

❖ Delirium: For a diagnosis of delirium by CAM, the patient must display: Presence of acute onset and fluctuating discourse and Inattention and either disorganized thinking or altered level of consciousness
❖ Hearing Impairment: defined as correctly hearing 6 or fewer of 12 numbers with both ears on a whisper test.
❖ Visual Impairment: defined as corrected binocular near vision worse than 20/70 on standard Jaeger test
❖ Current substance use: use of alcohol, khat, tobacco one or more in the past three months.
❖ Confusion Assessment Method scale: For a diagnosis of delirium by CAM, If features 1 and 2 and either 3 or 4 are present (CAM + /positive), a diagnosis of delirium is suggested [17].
❖ Richmond Agitation Sedation Scale: was used to assess the level of arousal and to indicate the delirium subtype of the psychomotor variety. Hyperactive delirium was defined as a patient’s RASS score falling between + 1 and + 4. Hypoactive delirium was defined as those with a RASS score between 0 and 3. Patients with mixed-type RASS scores at 0 and 3 were those who displayed both positive and negative RASS scores [18].
❖ Apache II score: acute physiology score + age points + chronic health points, higher scores correspond to more severe disease and higher risk of death [19].
❖ Charlson Comorbidity Index: this score consists of 21 variables and with a total score of 39 points score of 2, representing mild to moderate comorbidity, and a score of 8, representing severe comorbidity [20]

### Data collection tools and procedures

A structured interviewer-administered questionnaire was used to collect data. Questionnaires in this study about demographic and socioeconomic were developed after an extensive review of pieces of literature and similar study tools used in similar studies.

For the assessment of delirium, CAM was used which is validated for emergency settings. CAM has been translated into 10 languages where published articles are available. In literature from the ED, this tool has been cited to have a sensitivity of 94–100%, specificity of 90–95% and high inter-rater reliability. Several studies have been done to validate clinical usefulness. For a diagnosis of delirium by CAM, If features 1 and 2 and either 3 or 4 are present (CAM + /positive), a diagnosis of delirium is suggested [17].

In Sect. 2, DRS-R-98 is a 16-item observational clinician-rated scale with a maximum total severity score of 39 points was used to assess the severity of delirium and a total diagnostic score of 46 points showing sensitivity and specificity levels of 91% to 100% and 85% to 100% respectively. It is divided into two components. The first section has a 13-item severity scale that is utilized for continuous measurement throughout an episode of established delirium. A score of 12 points or more denotes complete delirium syndrome. The severity ratings vary from zero (no impairment) to 3 (severe impairment), and a severity score is > 15 points; it shows no severe delirium in the range of 12–15, less severe delirium in the range of 16–20, and severe delirium in the range of > 20 (severe delirium) [21].

For assessing cognitive status short portable mental status questionnaire (SPMSQ) was used, and the appropriate cut-off for SPMSQ was found to be 5 or more errors (sensitivity 78%, specificity 75%), regarding the scoring**:**0–2 errors: normal mental functioning,3–4 errors: mild cognitive impairment,5–7 errors: moderate cognitive impairment and 8 or more errors: severe cognitive impairment [22].

Furthermore, for assessing the level of arousal and to categorize the psychomotor subtype of delirium, RASS was used, a 10-point scale that scores from + 4 to -5. RASS was 84.0% sensitive (95% CI = 73.8% to 94.2%) and 87.6% specific (95% CI = 84.2% to 91.1%) for delirium. Patients will be evaluated for delirium by CAM if they will be responsive to verbal commands (a RASS score of other than -4 and -5), and patients with a RASS score between + 1 and + 4 will be considered to have hyperactive delirium. Patients with a RASS score between 0 and 3 were considered to have hypoactive delirium. Patients exhibiting both positive and negative RASS scores at 0 and 3 h were considered to have the mixed type [18].

In addition to assessing the severity of illness, Acute physiology and chronic health evaluation (APACHE II), Severity of Illness Scoring Systems was used, it consists of 12 variables with age points and chronic health points which gives a total score of 71 points and the total physiological derangement score is the sum of the individual scores (0–4), higher scores correspond to more severe disease and higher risk of death [19].

For the assessment of comorbidity, Charlson comorbidity index was used which consists of 21 variables and with total score of 39 points. A score of 2 represents mild to moderate comorbidity, and a score of 8 represents severe comorbidity.

### Data processing and analysis

After data collection was completed and the necessary information was obtained, data were checked for completeness. The study variable was coded in Epidata Manager Version 2.0.8.56 and data was entered, and edited by Epidata entry client version 2.0.7.22. Data were analyzed using SPSS version 20. For the analysis of obtained data simple descriptive statistics (mean, percentage, frequencies, and standard deviation) was used. Bivariate analysis was done to see the association of each independent variable with the outcome variable. Variables with a *p*-value ≤ 0.25 in bivariate analysis were entered into the multivariable logistic regression model to identify the effect of each independent variable on the outcome variables. Finally, a *p*-value of less than 0.05 was considered statistically significant, and an adjusted odds ratio with 95% CI was calculated to determine the strength of association.

### Data quality control

A pre-test was conducted on 24 participants (10% of the sample size) before the main study is done to identify impending problems in the proposed study and it was done one week before the day of actual data collection after training was given to the data collectors in Awetu primary hospital and a questionnaire translated into local language was used for data collection.

## Chapter 5: Result

### Socio-demographic and economic characteristics of respondents

Of the expected 422 respondents, 401 agreed to be enrolled in the study giving a response rate of 95%. Among 401 patients which participated in the study majority of them were males 65.8% (*n* = 264). The mean age of the respondents was 41.14(SD =  ± 15.92 years) with minimum and maximum ages ranging from 10 to 79 years respectively. Of the study participants enrolled in the study majority of them were Oromo by ethnicity 69.8% (*n* = 280), 60.9% (*n* = 241) of them were married, self-employed 56.6% (*n* = 227), and illiterate 33.7% (*n* = 135). Regarding the patient distribution within the emergency ward, most of the patients were in the surgical room 40.9% (*n* = 164) (Table 1).

**Table 1:** Socio- and demographic characteristics of participants among Jimma university medical center, emergency ward, August 2022 (*N* = 401)

Variable	Frequency (n)	Percentage %
Gender		
Male	264	65.8
Female	137	34.2
Age		
Less than 30	108	26.9
30–39	82	20.4
40–49	84	20.9
50–59	61	15.2
60–69	37	9.2
70–79	29	7.2
Ethnicity		
Oromo	280	69.8
Amhara	23	5.7
Tigre	2	0.5
Afar	3	0.7
Other	93	23.2
Marital status		
Married	241	60.1
Single	89	22.2
Divorced	44	11.0
Widowed	27	6.7
Economic Status		
< 2000	150	37.2
2001–2999	15	3.7
3000–4999	107	26.6
> 5000	31	7.7
Occupation		
Government Employed	65	16.2
Self-Employed	227	56.6
NGO	3	0.7
No Job	24	6.0
Private employed	82	20.4
Educational Status		
Illiterate	135	33.7
Primary School	114	28.4
Secondary School	86	21.4
Diploma and above	66	16.5
Emergency Ward		
Medical	132	32.9
Surgical	164	40.9
Resuscitation	105	26.2

The study also revealed that 16.2% (*n* = 65) of the study participants chewed khat at least once in their lifetime. Regarding drinking alcohol habit, 14.7% (*n* = 59) reported that they drink alcohol at least once in their lifetime while 6.7% (*n* = 27) were drinking alcohol over the last 30 days before the study. The study showed that 3.2% (*n* = 13) of the respondents smoked cigarettes at least once in their lifetime whereas 3.2% (*n* = 13) of the respondents have been smoking cigarettes in the past 30 days.

Furthermore, the study also revealed that 4% (*n* = 16) of participants had previous exposure to antipsychotics and also it was found from the research that 5.2% (*n* = 21) of study participants also were exposed to benzodiazepines. Regarding the history of polytherapy around 40.4% (*n* = 162) of the participants were on polytherapy.

In addition, 8.2% (*n* = 33) of study participants had a history of hearing impairment whereas 13.5% (*n* = 54) had a history of visual impairment. It is also found that 39.2% (*n* = 157), 82.3% (*n* = 330), 14.5% (*n* = 58) of the participants had bladder catheterization, intravenous fluid and frequent admission respectively (Table 2).

**Table 2:** Substance use, clinical and medication-related characteristics among patients admitted in emergency ward, Jimma medical center, August 2022 (*N* = 422)

Variable	Frequency(n)	Percent (%)
Alcohol use		
Lifetime		
Yes	59	14.7
No	342	85.3
Current use		
Before 1 month	32	8.0
During the past 1 month	27	6.7
Cigarette smoking		
Lifetime		
Yes	13	3.2
No	388	96.8
Current use		
Before 1 month	6	1.5
During the past 1 month	8	2.0
Khat use		
Lifetime		
Yes	65	16.2
No	336	83.8
Current use		
Before 1 month	31	7.7
During the past 1 month	35	8.7
Previous or current use of antipsychotics		
Yes	16	4
No	385	96
Previous or current use of benzodiazepines		
Yes	21	5.2
No	380	94.8
History of polytherapy		
Yes	162	40.4
No	239	51.6
Bladder catheterization		
Yes	157	39.2
No	244	60.8
Intravenous fluid		
Yes	330	82.3
No	71	17.7
Frequent Admission		
Yes	58	14.5
No	343	85.5
Previous Hearing impairment		
Yes	33	8.2
No	368	91.8
Previous Vision impairment		
Yes	54	13.5
No	347	86.5

### Prevalence of delirium

Approximately one-third (26.6%, *n* = 107) of the patients in the study had delirium. 3.0% (12) had mild delirium; 5.0% (*n* = 20) had moderate delirium, while 18.6% (*n* = 75) had severe delirium.

Furthermore, among patients who were found to be positive for delirium 10.9% (*n* = 44) had hyperactive subtype, while 14.8% (*n* = 60) had hypoactive delirium, and those who had mixed type were 0.7% (*n* = 3).

In addition regarding cognitive status, it was revealed from the study that 94(87.8%) had severe cognitive impairment, 10(9.3%) had moderate cognitive impairment, 6(5.6%) had mild cognitive impairment and none had normal mental functioning.

Of study participants with delirium 67(62.6%) of them had severe comorbidity, while 40(37.3%) had mild to moderate comorbidity.

### Factors associated with delirium

Socio-demographic and economic characteristics of patients like age, ethnicity, occupation and economic status didn’t show any association with delirium on bivariate analysis while sex, educational status, and sub-wards in the emergency ward were associated with delirium (Table 3).

**Table 3:** Bivariate analysis of socio-economic characteristics of study participants among Jimma medical center, August 2022. (*N* = 422)

Variable	**Delirium**		**COR &95%CI**	***P*-value**
	No (N%)	Yes (N%)		
Gender				
Male	178(44.39%)	81(20.2%)	2.030(1.232–3.347)	0.05*
Female	116(28.92%)	26(6.48%)	1	1
Age				
Less than 30	74(18.45%)	34(8.47%)	1	1
30–39	63(15.71%)	19(4.73%)	0.65(0.34–1.26)	0.207
40–49	69(17.2%)	15(3.74)	3.47(2.237–6.94)	0.034*
50–59	45(11.22%)	16(3.99%)	0.774(0.384–1.559)	0.473
60–69	24(5.98%)	13(3.24%)	1.179(0.536–2.592)	0.682
70–79	19(4.73%)	10(2.48%)	1.146(0.481–2.725)	0.759
Ethnicity				
Oromo	213(53.1%)	67(16.7%)	1.444(0.148–4.051)	0.533
Amhara	13(3.24)	10(2.49%)	1.880(0.736–4.805)	0.187
Tigre	1(0.25%)	1(0.25%)	0.769(0.455–1.300)	0.327
Afar	1(0.25%)	2(0.49%)	2.889(0.425–5.619)	0.203
Other	66(16.46%)	27(6.73%)	1	1
Marital status				
Married	180(44.88%)	61(15.21%)	1	1
Single	67(16.7%)	22(5.48%)	0.969(0.552–1.700)	0.912
Divorced	31(7.73%)	13(3.24%)	1.237(0.609–2.516)	0.556
Widowed	16(3.99%)	11(2.74%)	2.029(0.813–4.610)	0.091
Economic Status				
< 2000	107(26.68%)	49(12.21%)	1	1
2001–2999	16(3.99%)	2(0.49%)	2.486(1.317–4.692)	0.998
3000–4999	75(18.7%)	33(8.22%)	2.285(1.16–4.488)	0.016*
> 5000	22(5.48%)	9(2.24%)	2.097(0.87–5.381)	0.124
Occupation				
Government Employed	54(13.46%)	11(2.74%)	0.779(0.336–1.880)	0.560
Self-Employed	157(39.15%)	70(17.45%)	1.705(0.932–3.18)	0.083
NGO	2(0.49%)	1(0.25%)	1.912(0.163–2.357)	0.606
No Job	16(3.99%)	8(1.99%)	1.912(0.70–5.211)	0.205
Private employed	65(16.2%)	17(4.23%)	1	1
Educational Status				
Illiterate	88(21.9%)	47(11.72%)	1.984(0.497–3.948)	0.05*
Primary School	84(20.94%)	30(7.48%)	1.327(0.644–2.732)	0.443
Secondary School	70(17.45%)	16(3.99%)	0.894(0.381–1.893)	0.689
Diploma and above	52(12.96%)	14(3.49%)	1	1
Emergency Ward				
Medical	108(26.93%)	24(5.98%)	1	1
Surgical	124(30.92)	40(9.97%)	3.121(1.752–5.625)	0.003*
Resuscitation	62(15.46%)	43(10.72%)	1.452(0.823–2.562)	0.198

Bivirate analysis indicated that, khat chewing and Intravenous fluid were not significantly associated with delirium while cigarette smoking, hearing impairment, visual impairment, alcohol use, antipsychotic, bladder catheterization, benzodiazepine use and poly therapy were associated with delirium and entered to multivariable logistic regression model (Table 4).

**Table 4:** Bivariate analysis of Substance use, clinical and medication related charactertics among patients admitted in emergency ward, Jimma medical center, August 2022 (*N* = 422)

Variable	Delirium		COR &95%CI	*P*-value
	**No(N%)**	**Yes(N%)**		
Alcohol use				
Lifetime				
No	267(66.58%)	75(18.7%)	1	1
Yes	27(6.733%)	32(7.98%)	0.237(0.134–0.420)	0.001*
Current use				
No	21(20.8%)	11(2.74%)	1	1
Yes	6(1.49%)	21(5.23%)	6.68(2.08–3.201)	0.01
Cigaratte smoking				
Lifetime				
No	291(72.56%)	97(24.18%)	1	1
Yes	3(0.75%)	10(2.49%)	3.100(1.27–5.371)	0.001*
Current use				
No	6(1.49%)	1(0.25%)	1	1
Yes	3(0.75%)	3(0.75%)	2.052(1.060–3.972)	0.033*
Khat use				
Lifetime				
No	268(66.83%)	68(16.95%)	1	1
Yes	26(6.48%)	39(9.72%)	0.169(0.096–0.297)	0.004*
Current use				
No	17(4.24%)	14(3.49%)	1	1
Yes	10(2.49%)	25(6.23%)	0.410(0.287–0.585)	0.031*
Previous or current use of antipsychotics				
Yes	3(0.75%)	18(4.48%)	0.051(0.015–0.177)	0.013*
No	291(72.56%)	89(22.19)	1	1
Previous or current use of benzodiazepines				
Yes	3(0.75%)	18(4.48%)	0.051(0.15–0.77)	0.0043*
No	291(72.56%)	89(22.19%)	1	1
History of polytherapy				
Yes	70(17.45%)	92(22.94%)	0.051(0.028–0.094)	0.0023*
No	224(55.86%)	15(3.74)	1	1
Bladder catheterization				
Yes	80(19.95%)	77(19.2%)	0.146(0.089–0.239)	0.002*
No	214(53.36%)	30(7.48)	1	1
Intravenous fluid				
Yes	223(55.61%)	107(26.68)	0.23(0.874–1.652)	0.99
No	71(17.7)	0(0%)	1	1
Frequent Admission				
Yes	15(3.74%)	43(10.72)	0.080(0.042–0.153)	0.0021*
No	279(69.57%)	64(15.96%)	1	1
Hearing impairment				
Yes	14(3.49%)	19(4.73%)	0.232(0.112–0.481)	0.0033*
No	280(69.82%)	88(21.94%)	1	1
Vision impairment				
Yes	26(6.48%)	28(6.98%)	0.274(0.152–0.494)	0.0015*
No	268(66.83%)	79(19.7%)	1	1

From Multivariable logistic regression analysis it was found that current use of alcohol, visual impairment, frequent admission, bladder catheterization, and benzodiazepine exposure had significant association with delirium. In to their respective odd ratio, The odds of having delirium among patients with bladder catheterization was 7.7 fold higher in contrast to patients without bladder catheterization. The odds of participants with benzodiazepine exposure to had delirium were 7 times the odds of those without benzodiazepine exposure. Visual impairment was also associated with delirium; those participants with visual impairment were 2.7 times more likely to have delirium as compared with their counter parts current alcohol users 2.1 fold risk of having delirium than their counter parts and those participants who had frequent admission were 4.8 times more likely to have delirium as compared with their counter parts (Table 5).

**Table 5:** Multivariable analysis of factors associated with delirium among patients at Jimma medical center, August, 2022. *N* = 422

Variable	Delirium		AOR &95%CI	*P*-value
	**No (N%)**	**Yes (N%)**		
Alcohol use				
Current use				
No	21	11	1	1
Yes	6	21	0.216(0.086–0.542)	0.001*
Previous or current use of benzodiazepines				
Yes	3	18	6.503(1.57–29.558)	0.034*
No	291	89	1	1
Bladder catheterization				
Yes	80	77	7.746(3.752–15.993)	0.026*
No	214	30	1	1
Frequent Admission				
Yes	15	43	4.838(2.068–11.316)	0.013*
No	279	64	1	1
Vision impairment				
Yes	26	28	0.273(0.115–0.651)	0.004*
No	268	79	1	1

## Discussion

The study finding revealed that the prevalence of delirium among patients attending emergency department at Jimma medical center is 26.6%. This finding is comparable with a study done among old-age patients who underwent elective surgery in four teaching hospitals in Ethiopia which reported a prevalence of 27.6% [23].

Our finding is a bit lower than a study done among emergency unit of Jos university teaching hospital, Nigeria (35.9%) [7]. This could be due to sample size difference which was lower than ours, and they included patients admitted to the emergency setting due to accidents only, while our study included all types of cases that were admitted to the emergency setting.

The finding of our study is a bit higher than a study done in Egypt among older adults (> 65 years) [24]. The probable reason could be difference in sample size in which they used a larger sample size than ours. The study results are also higher than that of study done in Brazil( 17.9%) [25]. The possible reasons might be difference in study participants which had different sociodemographic and economic characteristics.

Regarding factors associated with delirium, our study finding showed that current use of alcohol, visual impairment, frequent admission, bladder catheterization, and benzodiazepine exposure had significant association with delirium.

From this study it was found that current use of alcohol was significantly associated with delirium. This might be explained by the fact that alcohol is the independent cause of delirium tremens further complications of chronic alcohol use like, chronic liver disease may have association with delirium. This finding is supported by a study conducted in Scotland which reported that alcohol-dependent patients had a significantly higher incidence of delirium than did those at low risk [26]. Additionally, a study from Norway which assessed the prevalence of delirium tremens among patients with alcohol use disorder reported that a lifetime encounter with DT was reported by 24% of the patients [27].

Visual impairment was also found to be significantly associated with delirium from this study, these findings were also supported by researches done in Ethiopia [28] and Chicago [29], some literatures propose sensory deprivation preceding delirium. Additionally, a study from Italy reported that the prevalence of visual impairment was higher in delirium patients [30].

Frequent admission was also found to be significantly associated with delirium from this study; these findings were also supported by researches done in Tanzania among medical inpatients [31] and Brazil [25].

Bladder catheterization was also found to be significantly associated with delirium from this study, these findings were also supported by researches done in Rwanda [32] and Belgium [33].

## Conclusion

Patients visiting Jimma Medical Center’s emergency room had a high rate of delirium. Contrary to its high occurrence, delirium appears to have avoidable causes, including current alcohol consumption, visual impairment, frequent hospitalization, bladder catheterization, and benzodiazepine exposure.

### Limitations

Recall bias was a significant drawback, and because this was a cross-sectional study, cause and effect couldn’t be established.

## Data Availability

The datasets used and/or analyzed during the current study are available from the corresponding author upon reasonable request.

## Abbreviations

CAM: Confusion Assessment Method scale
CAM-ICU: Confusion Assessment Method for the Intensive care unit
DRS-R-98: Delirium Rating Scale-Revised-98
ED: Emergency department
ICUs: Intensive care units
IQCODE: Informant Questionnaire on Cognitive Decline in the Elderly
JMC: Jimma Medical Center
LOS: Hospital length of stay
MMSE: Mini-Mental State Examination
MIS: Memory Impairment Screen
RASS: Richmond Agitation Sedation Scale
REMS: Rapid Emergency Medicine Score
WLM: Wechsler Logical Memory (WLM)
USD: United States Dollar
